# Incidence and clinical outcome of primary carcinomas of the major salivary glands: 10-year data from a population-based state cancer registry in Germany

**DOI:** 10.1007/s00432-022-04278-6

**Published:** 2022-08-22

**Authors:** Lisa Nachtsheim, M. Mayer, M. F. Meyer, F. Oesterling, H. Kajueter, C. Arolt, A. Quaas, J. P. Klussmann, P. Wolber

**Affiliations:** 1 Department of Otorhinolaryngology, Head and Neck Surgery, University of Cologne, Medical Faculty, Cologne, Germany; 2 Department of Otorhinolaryngology, Head and Neck Surgery, University Duisburg-Essen, Medical Faculty, Essen, Germany; 3 Cancer Registry North Rhine-Westphalia, Bochum, Germany; 4 Institute of Pathology, University of Cologne, Medical Faculty, Cologne, Germany

**Keywords:** Salivary gland cancer, Salivary gland neoplasms, Incidence, Survival, Epidemiology

## Abstract

**Purpose:**

The aim of this project was to provide an overview of the epidemiology of primary salivary gland carcinomas (SGC) in terms of incidence, distribution of clinicopathological features and survival in one of the largest cancer registries in Europe.

**Methods:**

Data were collected from patients with SGC of the major salivary glands registered in the population-based state cancer registry (Landeskrebsregister LKR) in North Rhine-Westphalia (NRW), Germany from 01/01/2009 to 12/31/2018. Age standardization of incidence was performed and relative survival estimates were computed by sex, histological group, age group and T-, N-, and M-stage.

**Results:**

A total of 1680 patients were included in this analysis. The most frequent tumor localization was the parotid gland (78%). Adenocarcinoma (not otherwise specified) was the most common tumor entity (18.5%). Most tumors were found in stages T1–T3 (29% T1; 29% T2; 28% T3). The age-standardized incidence rate (ASR) for SGC was 0.65/100,000 and remained stable during the observation period. There was an age-dependent incidence increasing especially from the age 70 years and onwards. The overall 5-year relative survival (RS) for all patients with SGC was 69.2%. RS was 80–95.6% for T1–2 stage tumors, 60.3% for T3, 47.3% for T4 stage, 87.4% for N0 and 51.2% for N1–2, 74.4% for M0 and 44.9% for M1.

**Conclusion:**

Age-standardized incidence for SGC has been stable for the observed 10-year period. Smaller tumors and those without lymph node or distant metastases had a better RS than more advanced tumors.

**Supplementary Information:**

The online version contains supplementary material available at 10.1007/s00432-022-04278-6.

## Introduction

Primary salivary gland carcinomas (SGC) are rare and account for approximately 3–6% of all head and neck malignancies (Guzzo et al. 2010; Mendenhall et al. 2005). Due to their heterogeneity regarding morphology, aggressiveness, and risk of recurrence the World Health Organization (WHO) divides the group of SGCs into more than 20 different entities (El-Naggar et al. 2017; Guzzo et al. 2010). The etiology of SGC remains unclear. Several risk factors such as age, radiation exposure and exposure to radioactive substances, prior cancer, chemicals and sawdust are being discussed (Lin et al. 2018).

There are only few studies with epidemiological data concerning SGC. Some of them focus only on specific tumor localizations (e.g., the parotid gland) or specific histological subtypes (Fu et al. 2019; Guntinas-Lichius et al. 2015; Hay et al. 2019; Rajasekaran et al. 2018; Westergaard-Nielsen et al. 2021).

The annual worldwide incidence rates of SGC range between 0.5 and 1.9/100,000 (Parkin et al. 2002). While studies in Asia and Europe have found stable incidences over a 10-year period (Westergaard-Nielsen et al. 2021; Fu et al. 2019), American studies revealed an increasing incidence (Carvalho et al. 2005; Del Signore and Megwalu 2017; Gupta et al. 2020).

In the current study, we aimed to provide an understanding of the incidence, demographic and oncological data of patients with primary SGC. We used data retrieved from the population-based state cancer registry in North Rhine-Westphalia (NRW), Germany, over a 10-year period between January 1st, 2009 and December 31st, 2018.

## Materials and methods

### Data retrieval

This study was based on data collected by the population-based state cancer registry (Landeskrebsregister; LKR) in NRW, Germany, over a 10-year period from January 1st, 2009, until December 31st, 2018. NRW is the most populated state of Germany, with nearly 18 million inhabitants (2020) and the LKR is one of the largest population-based cancer registries in Europe (Statistisches Bundesamt 2021). Malignant neoplasms diagnosed in NRW were reported to the cancer registry through pathology reports or clinical reports with a completeness of registration of over 90% (RKI 2019). Patients with tumor localization codes according to the International Classification of Diseases for Oncology (ICD-O-3) C07 (malignant neoplasm of parotid gland), C08.0 (malignant neoplasm of other and unspecified major salivary gland), C08.1 (malignant neoplasm of submandibular gland) and C08.9 (malignant neoplasm of major salivary gland, unspecified) were included in the analysis. A neoplasm that overlaps two or more adjoining regions and whose region of origin could not be determined was classified in the subcategory C08.8 (malignant neoplasm: overlapping lesion of major salivary gland). Squamous cell carcinomas, defined as ICD-O-3 morphology codes 8050/3–8084/3, were excluded from the analysis as information required for the differentiation between a primary and secondary squamous cell carcinomas was not sufficiently available in the state cancer registry data. Available tumor characteristics were tumor stage (T), nodal stage (N) and presence of distant metastases (M) pursuant to the current UICC Cancer Staging Classification at the time of diagnosis.

### Statistical analysis

Incidence rates are presented both as crude and age-standardized rates. Age standardization of incidence was performed according to the 1976 European Standard Population (1976 ESP) to allow comparison with international cohorts with a different underlying age structure. We, thus, refer to age-standardized incidence rate (ARS) in this analysis, not only crude incidence rates. Relative survival estimates by sex, tumor entity, 10-year age group and T-, N-, and M-stage were computed using the period approach (Brenner and Hakulinen 2009). Relative survival was defined as the ratio of the observed survival of cancer patients and expected survival in the general population given the same sex, age group and calendar period. We used sex- and calendar-year-specific life tables of the NRW population to estimate survival in the general population. Age-standardized relative survival estimates were derived using the International Cancer Survival Standards 1 (ICSS 1) standard population for cancer sites with increasing incidence by age (Corazziari et al. 2004). All relative survival analyses were computed with the periodR add-on package for R (Holleczek et al. 2009; Brenner and Hakulinen 2009). For explorative multivariate analyses, cox proportional hazards models with the endpoint of overall survival were computed. Only complete cases with respect to the included covariates were used. The proportional hazards assumption was assessed with a score test of the weighted residuals (Grambsch and Therneau 1994). The study was purely descriptive in nature and no a priori hypotheses were formulated. Therefore, no hypothesis tests and associated *p* values were computed. The precision of estimated incidence rates and relative survival proportions is expressed through 95% confidence intervals (95% CI).

The study was performed according to the regulations of the Ethics Committee of the University of Cologne.

## Results

### Demographics

In total, 2200 cases were registered. After exclusion of 520 cases of squamous cell carcinoma with unclear origin, 1680 patients with primary SGC remained for further analysis. Fifty-two percent (*n* = 868) of all patients were female and 48% (*n* = 812) male. The mean age at initial diagnosis was 65.3 years (see Table 1).

**Table 1:** Clinicopathologic characteristics of all salivary gland cancers in North Rhine-Westphalia (years of diagnosis 2009–2018)

	Women *n* = 812 (%^a^)	Men *n* = 868 (%)	Total *n* = 1680 (%)
Age (years)			
Mean	65.0	65.6	65.3
Median	67.5	68	68
Range	11 – 105	0 – 97	0 – 105
10th percentile	39	45	42
90th percentile	86	84	85
Primary site			
Parotid gland	627 (77.2)	685 (78.9)	1312 (78.1)
Submandibular glands	125 (15.4)	125 (14.4)	250 (14.9)
Sublingual glands	21 (2.6)	12 (1.4)	33 (2)
Major salivary glands, overlapping	4 (0.5)	5 (0.6)	9 (0.5)
Major salivary gland, NOS	35 (4.3)	41 (4.7)	76 (4.5)
Histological subgroup			
Other unspecific neoplasms	179 (22)	203 (23.4)	382 (22.7)
Adenocarcinomas NOS	116 (14.3)	194 (22.4)	310 (18.5)
Mucoepidermoid carcinoma	121 (14.9)	113 (13)	234 (13.9)
Adenoid cystic carcinoma	121 (14.9)	71 (8.2)	192 (11.4)
Acinic cell carcinoma	105 (12.9)	51 (5.9)	156 (9.3)
Carcinoma ex pleomorphic adenoma	21 (2.6)	39 (4.5)	60 (3.6)
Salivary duct carcinoma	14 (1.7)	45 (5.2)	59 (3.5)
Basal cell adenocarcinoma	31 (3.8)	20 (2.3)	51 (3)
Epithelial–myoepithelial carcinoma	28 (3.4)	21 (2.4)	49 (2.9)
Other rare morphologies	13 (1.6)	26 (3)	39 (2.3)
Myoepithelial carcinoma	17 (2.1)	16 (1.8)	33 (2)
Poorly differentiated—undifferentiated	11 (1.4)	12 (1.4)	23 (1.4)
Oncocytic carcinoma	3 (0.4)	14 (1.6)	17 (1)
Poorly differentiated—neuroendocrine carcinoma NOS	6 (0.7)	7 (0.8)	13 (0.8)
Polymorphic low-grade adenocarcinoma	7 (0.9)	6 (0.7)	13 (0.8)
Poorly differentiated—small cell carcinoma	5 (0.6)	7 (0.8)	12 (0.7)
Secretory carcinoma	5 (0.6)	5 (0.6)	10 (0.6)
Lymphoepithelial carcinoma	3 (0.4)	6 (0.7)	9 (0.5)
Carcinosarcoma NOS	2 (0.2)	5 (0.6)	7 (0.4)
Clear cell carcinoma NOS	3 (0.4)	3 (0.3)	6 (0.4)
Poorly differentiated—large cell neuroendocrine carcinoma	1 (0.1)	1 (0.1)	3 (0.2)
Poorly differentiated—large cell carcinoma NOS	–	3 (0.3)	2 (0.1)
Grading			
G1	79 (9.7)	44 (5.1)	123 (7.3)
G2	44 (5.4)	41 (4.7)	85 (5.1)
G3/G4	35 (4.3)	39 (4.5)	74 (4.4)
Grading unknown/not applicable^b^	654 (80.5)	744 (85.7)	1398 (83.2)
T-stage			
T1	180 (22.2)	114 (13.1)	294 (17.5)
T2	139 (17.1)	152 (17.5)	291 (17.3)
T3	114 (14)	170 (19.6)	284 (16.9)
T4	53 (6.5)	86 (9.9)	139 (8.3)
Unknown T-stage	326 (40.1)	346 (39.9)	672 (40)
N-stage			
N0	294 (36.2)	263 (30.3)	557 (33.2)
N1	48 (5.9)	58 (6.7)	106 (6.3)
N2	71 (8.7)	131 (15.1)	202 (12)
N3	8 (1)	21 (2.4)	29 (1.7)
Unknown N-stage	391 (48.2)	395 (45.5)	786 (46.8)
M-stage			
M0	233 (28.7)	277 (31.9)	510 (30.4)
M1	16 (2)	22 (2.5)	38 (2.3)
Unknown M-stage	563 (69.3)	569 (65.6)	1132 (67.4)
UICC-stage			
UICC I	69 (8.5)	39 (4.5)	108 (6.4)
UICC II	48 (5.9)	48 (5.5)	96 (5.7)
UICC III	52 (6.4)	50 (5.8)	102 (6.1)
UICC IV A	49 (6)	97 (11.2)	146 (8.7)
UICC IV B	5 (0.6)	22 (2.5)	27 (1.6)
UICC IV C	16 (2)	22 (2.5)	38 (2.3)
Unknown stage	573 (70.6)	590 (68)	1163 (69.2)
Deaths			
Cause of death ICD-10 C07/C08	167 (20.6)	198 (22.8)	365 (21.7)
Other causes of death	119 (14.7)	180 (20.7)	299 (17.8)

Percentages have been rounded and may not reach 100%. The percentages on T-, N- and M-stage refer to those patients with available data.

^a^Column percentages.

^b^Histopathologic grading was assumed to be applicable only for mucoepidermoid carcinoma and adenoid-cystic carcinoma

### Tumor characteristics

Seventy-eight percent (*n* = 1312) of the patients were diagnosed with SCG of the parotid gland, 15% had a carcinoma of the submandibular gland (*n* = 250). Two percent had a carcinoma of the sublingual gland (*n* = 33), 4.5% (*n* = 76) had an unspecified carcinoma of the major salivary glands and 0.5% (*n* = 9) had an overlapping lesion of major salivary glands. In total, 22 different tumor entities were reported (Table 1).

The most frequently diagnosed entity was adenocarcinoma not otherwise specified (ANOS), followed by mucoepidermoid carcinoma (MuEp) and adenoid-cystic carcinoma (ACC). The most frequent tumor entity in the sublingual gland was ACC. The detailed distribution of the entities and their relation to the different salivary glands is displayed in Tables 1 and 2.

**Table 2:** Distribution of morphologic group in relation to different salivary glands

Histological subgroup	Parotid gland *n* (%)^a^	Submandibular gland *n* (%)	Sublingual gland *n* (%)	Salivary glands overlapping *n* (%)	Unspecified *n* (%)
Adenoid cystic carcinoma	89 (46.4)	80 (41.7)	10 (5.2)	2 (1.0)	11 (5.7)
Other rare morphologies	32 (82.1)	6 (15.4)	0 (0.0)	0 (0.0)	1 (2.6)
Adenocarcinoma NOS	237 (76.5)	54 (17.4)	6 (1.9)	2 (0.6)	11 (3.5)
Acinic cell carcinoma	144 (92.3)	8 (5.1)	2 (1.3)	0 (0.0)	2 (1.3)
Basal cell adenocarcinoma	42 (82.4)	3 (5.9)	3 (5.9)	0 (0.0)	3 (5.9)
Epithelial–myoepithelial carcinoma	45 (91.8)	1 (2.0)	0 (0.0)	0 (0.0)	3 (6.1)
Poorly differentiated—large cell neuroendocrine carcinoma	2 (66.7)	1 (33.3)	0 (0.0)	0 (0.0)	0 (0.0)
Poorly differentiated—large cell carcinoma NOS	2 (100)	0 (0.0)	0 (0.0)	0 (0.0)	0 (0.0)
Poorly differentiated—small cell carcinoma	12 (100)	0 (0.0)	0 (0.0)	0 (0.0)	0 (0.0)
Poorly differentiated—neuroendocrine carcinoma NOS	9 (69.2)	4 (30.8)	0 (0.0)	0 (0.0)	0 (0.0)
Poorly differentiated—undifferentiated	18 (78.3)	3 (13.0)	0 (0.0)	0 (0.0)	2 (8.7)
Carcinoma ex pleomorphic adenoma	47 (78.3)	9 (15.0)	0 (0.0)	0 (0.0)	4 (6.7)
Carcinosarcoma NOS	7 (100)	0 (0.0)	0 (0.0)	0 (0.0)	0 (0.0)
Clear cell carcinoma NOS	4 (66.7)	2 (33.3)	0 (0.0)	0 (0.0)	0 (0.0)
Lymphoepithelial carcinoma	8 (88.9)	1 (11.1)	0 (0.0)	0 (0.0)	0 (0.0)
Mucoepidermoid carcinoma	194 (82.9)	26 (11.1)	7 (0.0)	0 (0.0)	7 (3.0)
Myoepithelial Carcinoma	29 (87.9)	3 (9.1)	0 (0.0)	0 (0.0)	1 (3.0)
Oncocytic carcinoma	14 (82.4)	2 (11.8)	0 (0.0)	0 (0.0)	1 (5.9)
Polymorphic low-grade adenocarcinoma	8 (64.5)	0 (0.0)	1 (7.7)	2 (15.4)	2 (15.4)
Secretory carcinoma	9 (90.0)	1 (10.0)	0 (0.0)	0 (0.0)	0 (0.0)
Other unspecific neoplasms	310 (81.2)	40 (10.5)	4 (1.0)	3 (0.8)	25 (6.5)
Salivary duct carcinoma	50 (84.7)	6 (10.2)	0 (0.0)	0 (0.0)	3 (5.1)

^a^Row percentages

The following percentages refer to those patients with available data on T-, N- and M-stage. Most of the patients were diagnosed with a tumor stages T1–3 (see Table 1) and only 14% in stage T4 (Table 1). In 672 patients, data on T-stage was missing. Lymph node metastases corresponded to N1 stage in 12%, N2 stage in 23% and to N3 stage in 3%. Sixty-two percent of all patients had no signs of lymph node metastases. Information on nodal status was missing in 786 patients. Seven percent of the patients had distant metastases without available information on the exact localization. In 1,132 patients, data on M-stage was missing. For patients under the age of 18 (1.1%), all carcinomas were stage T1 or T2 and no lymph node or distant metastases were reported. Out of all tumor entities, salivary duct carcinomas (SDC) had the highest rate of distant metastases (6.8%; Table 3).

**Table 3:** T-, N- and M-stage distribution of the five most frequent salivary gland carcinomas in NRW

T-/N-/M-stage	Acinic cell carcinoma *n* (%)^a^	Adenocarcinoma NOS *n* (%)	Adenoid cystic carcinoma *n* (%)	Mucoepidermoid carcinoma *n* (%)	Salivary duct carcinoma *n* (%)
T-stage					
T1	44 (28.2)	47 (15.2)	39 (20.3)	79 (33.8)	13 (22)
T2	50 (32.1)	52 (16.8)	34 (17.7)	55 (23.5)	10 (16.9)
T3	16 (10.3)	80 (25.8)	49 (25.5)	39 (16.7)	13 (22)
T4	6 (3.8)	39 (12.6)	20 (10.4)	21 (9)	10 (16.9)
TX	40 (25.6)	92 (29.7)	50 (26)	40 (17.1)	13 (22)
N-stage					
N0	79 (50.6)	90 (29)	86 (44.8)	122 (52.1)	16 (27.1)
N1	7 (4.5)	32 (10.3)	18 (9.4)	16 (6.8)	6 (10.2)
N2	16 (10.3)	70 (22.6)	18 (9.4)	26 (11.1)	20 (33.9)
N3	1 (0.6)	9 (2.9)	2 (1)	1 (0.4)	3 (5.1)
NX	53 (34)	109 (35.2)	68 (35.4)	69 (29.5)	14 (23.7)
M-stage					
M0	43 (27.6)	113 (36.5)	79 (41.1)	96 (41)	21 (35.6)
M1	5 (3.2)	10 (3.2)	5 (2.6)	2 (0.9)	4 (6.8)
MX	108 (69.2)	187 (60.3)	108 (56.2)	136 (58.1)	34 (57.6)

^a^Column percentages

### Incidence

The age-standardized incidence rate (ASR) for all entities was 0.64/100,000. When divided into the different tumor localizations, the ASR was 0.50/100,000 for parotid gland carcinomas, 0.10/100,000 for carcinomas of the submandibular gland, and 0.01/100,000 for carcinomas of the sublingual gland (Supplementary Table 1). The mean ASR was higher in male (0.72/100,000) than in female patients (0.59/100,000) (Supplementary Tables 1 and 2). There was a continuous increase of incidence with advancing age when further looking at age group specific incidence (Supplementary Table 1). Incidence rates peaked in patients > 90 years of age (4.70/100,000), whereas the lowest incidence was found in children (0.063/100,000). The detailed distribution on ARS regarding tumor and nodal stage as well as distant metastases is displayed in Supplementary Table 1.

**Fig. 1:**
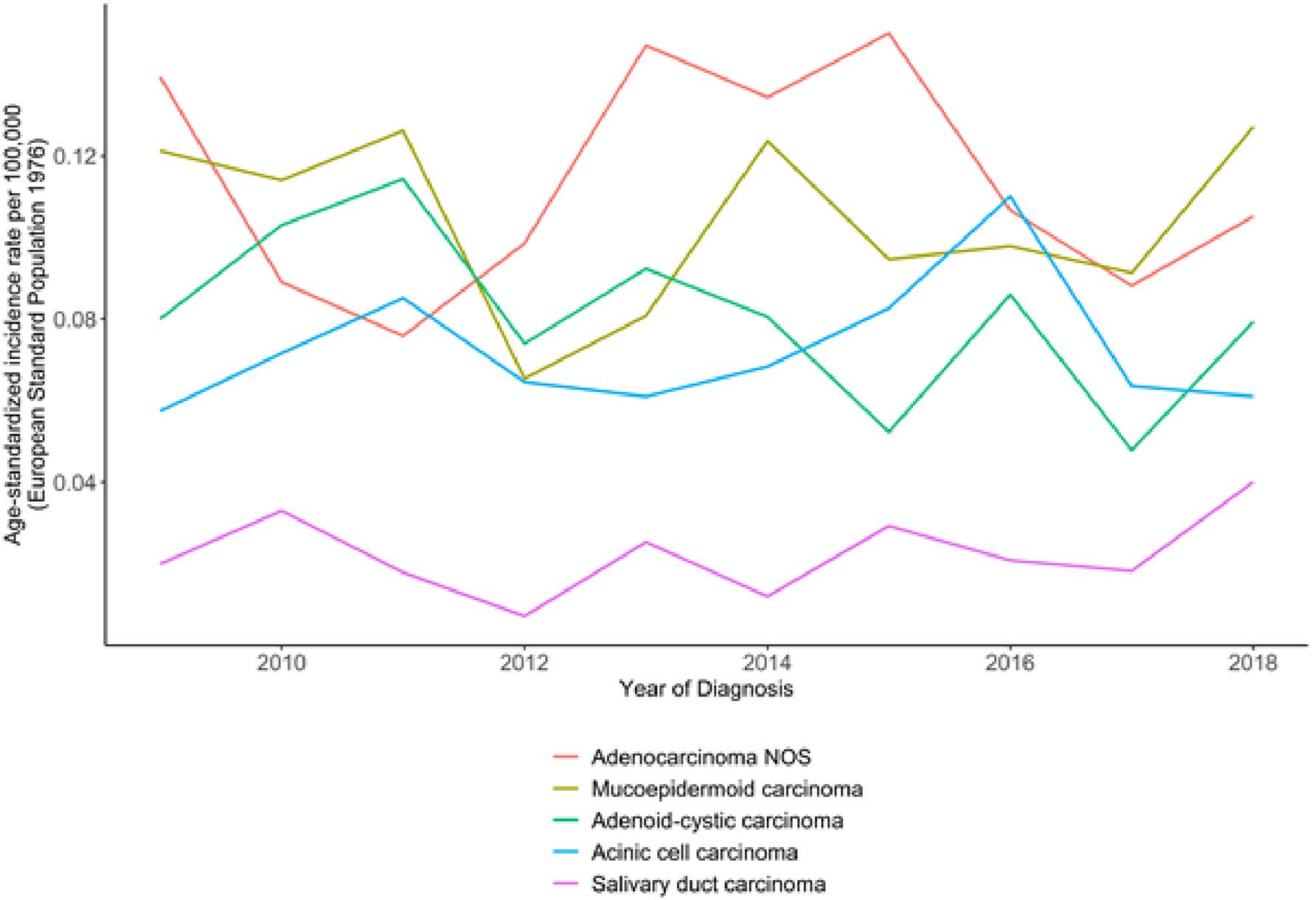
Age-standardized incidence rates of the five most frequent histologic subgroups of major salivary gland cancers in NRW. Years of diagnosis 2009–2018

Regarding the five most frequent tumor entities, the ASR for ANOS was 0.11/100,000, for MuEp 0.1/100,000, for ACC 0.08/100,000, for Acinic cell carcinoma (Acin) 0.7/100,000 and for SDC 0.02/100,000 (Supplementary Table 1, Fig. 1). There was an overall stable incidence in all morphologic groups (Fig. 1). The incidence of secretory carcinoma increased between 2013 and 2018 from 0.005/100,000 to 0.011/100,000. When correlated with T-, N- and M-stage and sex, we also found stable incidences over the 10-year period.

### Survival

**Fig. 2:**
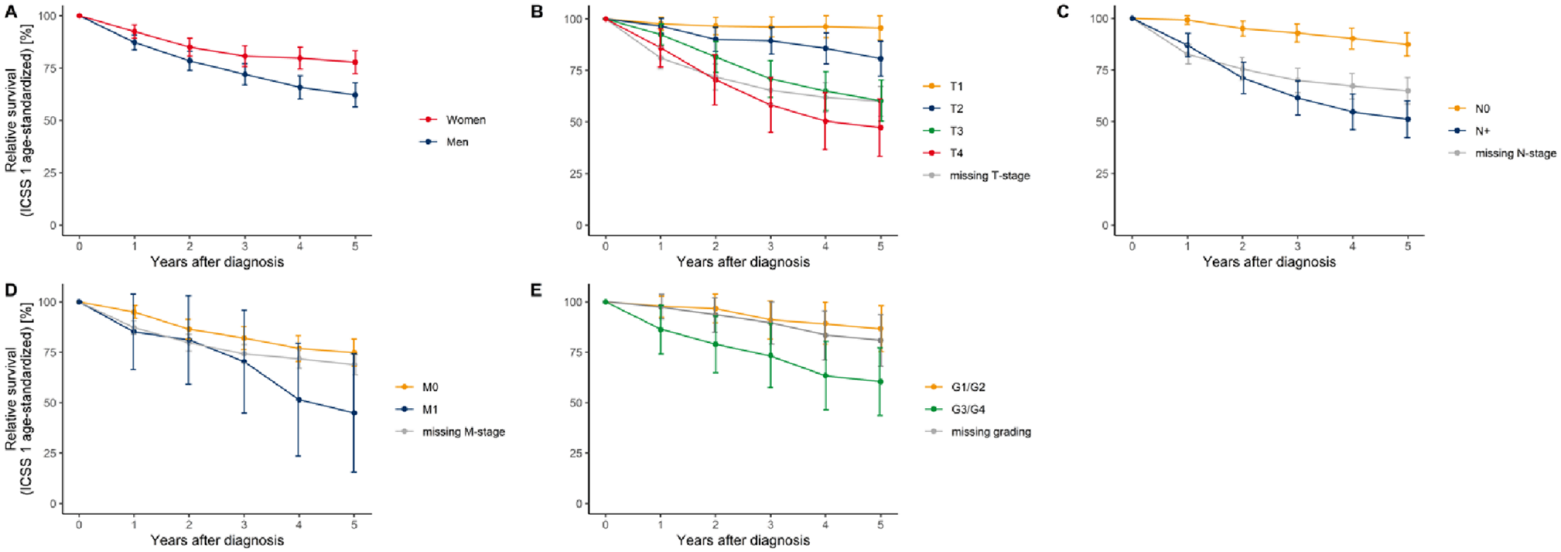
Age-standardized relative survival of salivary gland cancer patients diagnosed between 2009 and 2018 in NRW using the period approach; stratified by sex (**A**), tumor size at diagnosis (**B**), lymph node status at diagnosis (**C**), presence of distant metastasis at diagnosis (**D**) and histopathologic grading (**E**; only mucoepidermoid carcinoma and adenoid-cystic carcinoma cases)

**Fig. 3:**
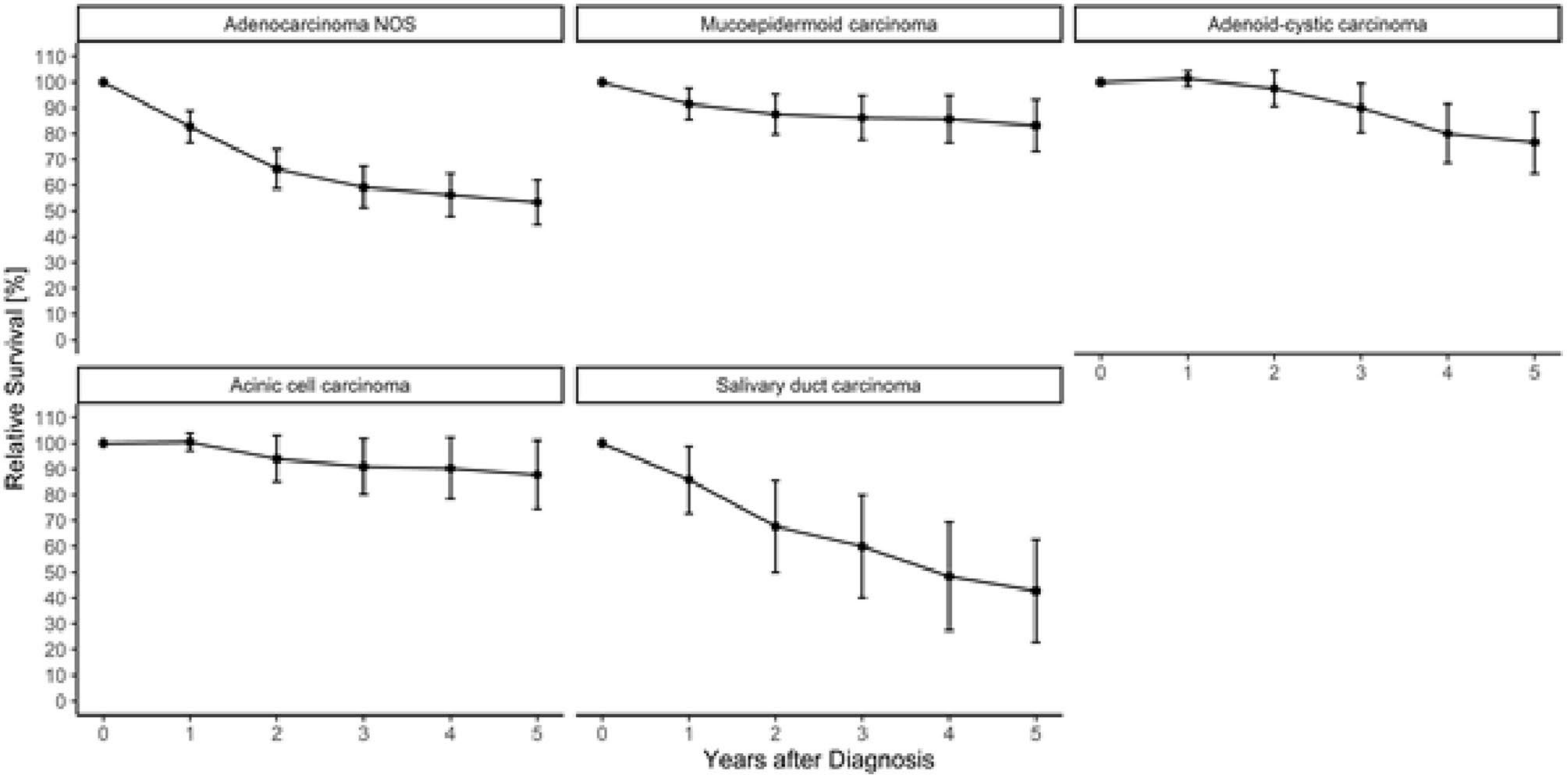
Age-standardized relative survival of the five most frequent histologic subgroups of major salivary gland cancers in NRW using the period approach (calendar period 2014–2018). Years of diagnosis 2009–2018. Age-standardized using the ICSS 1 standard population

Table 4 displays the 5-year relative survival (RS). RS for all patients was 69.2%. Female patients had a better RS than male patients. RS was higher for lower T-stages than for advanced tumors (Table 4; Fig. 2). Patients without cervical lymph node metastases had a higher RS than patients with lymph node metastases (87.4% vs. 51.2%). Furthermore, patients with distant metastases had a lower RS than those without (44.9% vs. 74.8%). When subdivided into entities, the highest RS rates were found in Acin, basal cell adenocarcinoma and MuEp. The lowest RS rate was found in SDC and ANOS (Table 4; Fig. 3). ACCs had a RS rate of 79%. For 21% of the patients, SGC was reported as cause of death (Table 1).

**Table 4:** Relative 5-year survival proportions (in percent) using the period approach

	Relative 5-year survival estimate	Std. error	95% confidence interval
Overall	69.2	2.1	[65.1; 73.3]
Sex			
Men	62.1	2.9	[56.4; 67.9]
Women	77.8	2.8	[72.3; 83.3]
T-stage			
T1	95.6	3.0	[89.8; 101.5]
T2	80.8	4.3	[72.3; 89.2]
T3	60.3	5.1	[50.4; 70.3]
T4	47.3	7.1	[33.3; 61.3]
TX	60.0	3.7	[52.6; 67.3]
N-stage			
N0	87,4	2.9	[81.8; 93.0]
N +	51.2	4.5	[42.3; 60.0]
NX	65.0	3.3	[58.5; 71.4]
M-stage			
M0	74,8	3.5	[68.0; 81.6]
M1	44.9	14.9	[15.7; 74.2]
MX	68.8	2.6	[63.8; 73.9]
Histological subgroup			
Acinic cell carcinoma	90.4	4.7	[81.2; 99.6]
Mucoepidermoid carcinoma	85.6	4.3	[77.1; 94.1]
Adenoid cystic carcinoma	79.9	5.1	[69.9; 89.9]
Adenocarcinoma NOS	53.3	4.5	[44.4; 62.2]
Salivary duct carcinoma	43.3	10.6	[22.5; 64.1]

Estimates for the complete cohort and stratified by sex, T-, N- and M-Stage and histological subgroup

To investigate whether the survival difference between males and females was due to different stage or age distributions between the sexes, we additionally calculated Cox proportional hazards models. The globas score test for proportional hazards did not indicate a violation of the proportional hazards assumption (*Χ*^2^ = 4.58, *df* = 7, *p* = 0.71). The unadjusted model resulted in a hazard ratio for men compared to women of 1.9 (95% CI [1.60; 2.19]). The hazard ratio for male sex after adjusting for T-, N- and M-stage and age at diagnosis was 1.74 (95% CI [1.44; 2.04]).

## Discussion

This is one of the largest population-based studies on the incidence and survival of SGCs in Europe. We provide an overview of the incidence and stage distribution of SGCs over a 10-year period as well as demographic data including survival rates.

The overall ASR for all histologic subtypes was 0.649/100,000 and remained stable over the 10-year period. There are significant geographic variations concerning the incidence for SGC. While European studies have reported a stable age-standardized incidence of 0.9/100,000 over a 26-year period (1990–2015) (Westergaard-Nielsen et al. 2021), other American studies have found a slight increase from 1.0 to 1.1/100,000 (1974–1990) (Carvalho et al. 2005) and 0.7 to 1.2/100,000 (1973–2015) (Gupta et al. 2020). In Asian studies, a stable age-standardized incidence of 0.79/100,000 over a 10-year period has been observed (Fu et al. 2019). Since etiological and risk factors for SGC are mainly unknown, it is difficult to draw any conclusions from these variations of incidence. Some authors explain the increasing incidence in the US with advances in early detection through improved diagnostic tests, increasing awareness and progress concerning diagnostic imaging (Gupta et al. 2020; D'heygere et al. 2018).

We report an incidence of SGCs increasing with age, with the highest age group specific incidence in the group of patients over 90 years. Guntinas-Lichius et al. (2015) found similar results with an increase of the crude incidence for malignant parotid tumors especially in the age group over 85 years. In our study, only 1.1% of the patients were under the age of 18. With an incidence of 0.06/100,000, these patients showed the lowest incidence of all age groups. SGCs in children are very rare and less than 5% of all SGC occur in children (Luna et al. 1991).

As reported by other authors, we could confirm the finding of lower tumor stages with no lymphatic or distant metastases in children, as well as MuEp being the most frequently diagnosed entity in children (Gontarz et al. 2018; Janz et al. 2018).

Age is a well-known risk factor for general cancer development through the accumulation of mutations over a lifetime. Furthermore, extrinsic (e.g., alcohol or smoking) and intrinsic (cell division) factors influence general carcinogenesis (Laconi et al. 2020). However, specific risk factors for SGCs remain uncertain. Various factors such as specific occupational and industrial-related exposures, radiation and prior cancer are being discussed (Radoï et al. 2018). Due to the large variety of morphologically different tumor entities and the low incidence of SGC, research concerning the specific etiology still presents a challenge.

The overall ASR was higher in male patients than in female patients, which is consistent with the findings of other authors when looking at crude incidences (Fu et al. 2019; Luukkaa et al. 2005, Guntinas-Lichius et al. 2015). This observation has been attributed to occupational risk factors in male dominated jobs (Boukheris et al. 2009).

The most frequent tumor localization for SGC was the parotid gland where more than 75% of the tumors were located. Fu et al. localized 54%, Luukkaa found 64% and Westergaard et al. found 51.8% of all SGC in the parotid gland, but all these studies included minor salivary gland carcinomas in the calculation, which changes this proportion (Luukkaa et al. 2005; Westergaard-Nielsen et al. 2021; Fu et al. 2019). About 15% of the tumors in this analysis were located in the submandibular gland and 2% in the sublingual gland. Interestingly, we found that ACCs were almost equally located in the parotid (46.4%) and submandibular gland (41.7%), making them the most frequent histologic subtype for submandibular SGC in this analysis. In a study from Finland, authors found that the localization of ACCs was also equally distributed over the parotid, submandibular and sublingual gland, while a study from Denmark reported ACCs primarily in the minor salivary glands (Luukkaa et al. 2005; Therkildsen et al. 1998).

In the current study, the most frequent entity was ANOS. While this finding is consistent with the results reported by some authors (Guzzo et al. 2010; Guntinas-Lichius et al. 2015; Fu et al. 2019), others found ACC (Westergaard-Nielsen et al. 2021; Luukkaa et al. 2005) or MuEp (Boukheris et al. 2009; Xiao et al. 2016; Gupta et al. 2020) as the most frequent SGC subtypes. However, some authors included exclusively parotid gland cancer in their analysis (Guntinas-Lichius et al. 2015; Xiao et al. 2016) and others included both major and minor salivary glands (Fu et al. 2019; Westergaard-Nielsen et al. 2021) which can distort this frequency distribution. Additionally, it must be noted that ANOS is a diagnosis of exclusion. During the observed 10-year period, not only diagnostic criteria developed and new entities were defined, but also new diagnostic tools were established (Haller et al. 2020; Rooper et al. 2021). Therefore, ANOS are nowadays becoming a lesser diagnosed entity since modern techniques allow more specific classification, and thus more recent studies will most likely find fewer cases of ANOS (Rooper et al. 2021).

SDC showed the highest incidence of lymph node and distant metastases and the lowest RS. SDCs rank among the most aggressive SGC subtypes and are characterized by high rates of metastases and recurrence (Nakaguro et al. 2020). Furthermore, it is associated with low survival. However, when a 5-year disease-free interval is completed, survival rates stabilize (Schmitt et al. 2017; D'heygere et al. 2018).

Secretory carcinoma is a relatively new tumor entity, first described in 2010 by Skalova et al. and accepted as a new WHO SGC subtype in 2017 (Skálová et al. 2010; Ihrler et al. 2018). Prior to that, it was frequently classified as Acin. In our analysis, cases of secretory carcinoma before 2017 have possibly been reclassified. Evidently, since this study relied on cancer registry data, paraffin blocks or slides were not available, rendering a retrospective revision of the diagnoses impossible. Over the past decade, many new distinct tumor entities have been introduced. Accordingly in our cohort, we witnessed an increase of secretory carcinoma diagnoses over the past years. Future studies like the current one will reveal the epidemiologic impact of the most recent diagnostic advances in the field of salivary gland pathology.

Moreover, we observed an even distribution of early T-stages, in particular T1–3. This is in line with data from another European study, in which about two thirds of the SGCs were diagnosed in stage T1 or T2 (Westergaard-Nielsen et al. 2021). Guntinas-Lichius et al. (2015) reported an increase of stage T4 tumors between 1996 and 2011, which could not be confirmed in the current study.

Lymph node metastases were found in 38% of the patients in this analysis. While Klussmann et al. have reported similar results (38%), Xiao et al. found 24.4% and Westergaard et al. 18.1% nodal metastases in their studies (Xiao et al. 2016; Klussmann et al. 2008; Westergaard-Nielsen et al. 2021). Only 7% of the patients had distant metastases in this analysis. Most authors found a comparable low rate of 1.7–5% (Westergaard-Nielsen et al. 2021; Guntinas-Lichius et al. 2015).

The RS rate for all entities was 69%. Patients with absence of pathological lymph nodes had an RS rate of 87%. RS rate of patients in stage T1 was 97% and dropped to 47% for T4 tumors. Other authors have also shown that the RS varies in a wide range depending on disease stage when comparing localized, regional and distant disease (Gupta et al. 2020; Westergaard-Nielsen et al. 2021). Furthermore, variations in survival rates can be explained by differences in parameters such as age, proportion of histologic subtypes and anatomical sites (Westergaard-Nielsen et al. 2021).

In our analysis, the RS rate was as low as 44.9% in presence of distant metastases. Hematogenous metastases from SGC are most frequently found in the lungs (80%), bones (15%), liver and other sites (5%) and have been identified as the main cause of tumor related death (Guzzo et al. 2010). ACC and SDC showed the highest rate of distant metastases and SDC had the lowest RS rate of all histologic subgroups. ACCs are known to grow slowly, which explains the long period between the occurrence of distant metastases and death (Guzzo et al. 2010). Therefore, in contrast to SDC, the RS rate of ACC (80%) and MuEp (86%) in this analysis was comparatively high. Prognosis and survival of patients with MuEp depends largely on tumor grading. While low-grade tumors have reported survival rates of up to 92–100%, high-grade tumors are more prone to develop lymphatic and hematogenous metastases with survival rates of up to 43% (Guzzo et al. 2010; Seethala 2009). We also observed a better survival in patients with tumor grading G1–2 than in those with G3–4. In this analysis, grading was analyzed only for MuEp and ACC since these entities can be graded using a standardized grading system with reliable reproductivity (Seethala 2009).

Furthermore, we observed a better RS for women than men. With an adjusted hazard ratio for males of 1.74, an additional Cox regression model revealed that this difference could not be explained by differences in age, T-, N- or M-stage alone. One explanation for the poorer survival in male patients in this current analysis could be the higher incidence of SDC in men as well as confounding from unobserved variables such as comorbidities or smoking behavior, especially because overall survival and not disease-specific survival was considered. Other authors have also identified male sex as a prognostic factor associated with SGC outcome (Westergaard-Nielsen et al. 2021; Luukkaa et al. 2005).

There are several limitations to this study. First, there was a relatively high number of missing or incomplete oncological data from the state cancer registry. Second, the study is limited by its retrospective design and its observational aspect. Additionally, no data on therapy were available. Of note, we excluded minor salivary gland tumors in this analysis out of technical reasons. Minor salivary gland tumors are not categorized under the common ICD-codes for SGC but according to localization (e.g., oral cavity, mouth base, etc.). Additionally, we chose to exclude squamous cell carcinomas in our analysis since the differentiation between primary and secondary squamous cell carcinoma of salivary glands can only be made after exclusion of prior cutaneous squamous cell carcinoma according to the WHO. This information is mostly not accessible for a cancer registry (Oesterling et al. 2022). However, through the exclusion of squamous cell carcinoma from our cohort we gained purer data and avoided selection bias of falsely classified SGCs.

In this large population-based cancer registry study, we showed that the incidence of SGCs remained stable over a 10-year period and increased with advanced age. Tumors mainly affected the parotid gland with ANOS being the most frequent histologic subtype. Most tumors were diagnosed in low tumor stages, which were shown to have a better RS, however, depending on histologic subtype and presence of lymph node or distant metastases.

## Supplementary Information

Below is the link to the electronic supplementary material.

Supplementary file1 (DOCX 19 kb)

Supplementary file2 (DOCX 13 kb)
