# Supplementary material for: Incidence and clinical outcome of primary carcinomas of the major salivary glands: 10-year data from a population-based state cancer registry in Germany
Source: J Cancer Res Clin Oncol. 2022 Aug 22;149(7):3811–21. doi: 10.1007/s00432-022-04278-6 (PMC10314868; doi:10.1007/s00432-022-04278-6)
Supplement: Supplementary file 1 — Supplementary file1 (DOCX 19 kb) [file 432_2022_4278_MOESM1_ESM.docx]

**Supplementary Table 2** Cox proportional hazards models for the endpoint of overall survival in salivary gland carcinoma patients in NRW. Only cases with complete covariate information (n = 510). Model 1: Estimating the hazard ratio of male compared to female sex; Model 2: Estimating the hazard ratio of male compared to female sex, adjusted for T-, N- and M-stage and age at diagnosis.

| **Estimate** | **Hazard Ratio** | **Std. error** | **95% Confidence interval** |
| --- | --- | --- | --- |
| **Unadjusted Model** |  |  |  |
| **Sex** |  |  |  |
| Women (Reference) | 1 | - | - |
| Men | 1.90 | 0.15 | [1.60; 2.19] |
| **Adjusted Model** |  |  |  |
| **Sex** |  |  |  |
| Women (Reference) | 1 | - | - |
| Men | 1.74 | 0.15 | [1.44; 2.04] |
| **T-Stage** |  |  |  |
| T1 (Reference) | 1 | - | - |
| T2 | 1.61 | 0.26 | [1.09; .2.13] |
| T3 | 2.04 | 0.26 | [1.54; 2.54;] |
| T4 | 3.56 | 0.26 | [3.05; 4.07] |
| **N-Stage** |  |  |  |
| N0 (Reference) | 1 | - | - |
| N+ | 2.22 | 0.16 | [1.92; 2.53] |
| **M-Stage** |  |  |  |
| M0 (Reference) | 1 | - | - |
| M1 | 1.37 | 0.23 | [0.91; 1.82] |
| **Age at Diagnosis** | 1.04 | 0.01 | [1.03; 1.06] |
|  |  |  |  |
