# Supplementary material for: Incidence and clinical outcome of primary carcinomas of the major salivary glands: 10-year data from a population-based state cancer registry in Germany
Source: J Cancer Res Clin Oncol. 2022 Aug 22;149(7):3811–21. doi: 10.1007/s00432-022-04278-6 (PMC10314868; doi:10.1007/s00432-022-04278-6)
Supplement: Supplementary file 2 — Supplementary file2 (DOCX 13 kb) [file 432_2022_4278_MOESM2_ESM.docx]

**Supplementary Table 1.** Mean annual incidence rates for primary major salivary gland cancers diagnosed between 2009 and 2018 in NRW. Crude and age-standardized estimates (1976 European Standard Population).

|  | **Crude incidence rate/100.000** | **95% Confidence interval** | **Age-standardized incidence rate/100.000** | **95% Confidence interval** |
| --- | --- | --- | --- | --- |
| **Overall** | 0.945 | [0.900; 0.991] | 0.649 | [0.616; 0.683] |
| **Sex** |  |  |  |  |
| Men | 1.000 | [0.933; 1.066] | 0.723 | [0.673; 0.773] |
| Women | 0.894 | [0.832; 0.955] | 0.599 | [0.553; 0.644] |
| **Age-group-specific incidence** |  |  |  | - |
| < 18 | 0.063 | [0.035; 0.092] | - | - |
| 18 – 29 | 0.188 | [0.135; 0.241] | - | - |
| 30 – 39 | 0.341 | [0.262; 0.419] | - | - |
| 40 – 49 | 0.561 | [0.472; 0.651] | - | - |
| 50 – 59 | 1.044 | [0.922; 1.166] | - | - |
| 60 – 69 | 1.635 | [1.457; 1.813] | - | - |
| 70 – 79 | 2.449 | [2.216; 2.682] | - | - |
| 80 – 89 | 3.418 | [3.026; 3.809] | - | - |
| ≥ 90 | 4.80 | [3.649; 5.946] | - | - |
| **Primary site** |  |  |  |  |
| Parotid gland | 0.738 | [0.698; 0.778] | 0.502 | [0.473; 0.531] |
| Submandibular glands | 0.141 | [0.123; 0.158] | 0.102 | [0.088; 0.115] |
| Sublingual glands | 0.019 | [0.012; 0.025] | 0.014 | [0.009; 0.018] |
| Major salivary glands, overlapping | 0.005 | [0.002; 0.008] | 0.004 | [0.001; 0.006] |
| Major salivary gland, NOS | 0.043 | [0.033; 0.052] | 0.028 | [0.022; 0.035] |
| **T-Stage** |  |  |  |  |
| T1 | 0.165 | [0.147; 0.184] | 0.130 | [0.114; 0.146] |
| T2 | 0.160 | [0.141; 0.178] | 0.109 | [0.096; 0.123] |
| T3 | 0.164 | [0.145; 0.183] | 0.120 | [0.106; 0.135] |
| T4 | 0.078 | [0.065; 0.091] | 0.049 | [0.040; 0.058] |
| TX | 0.378 | [0.350; 0.407] | 0.240 | [0.221; 0.260] |
| **N-Stage** |  |  |  |  |
| N0 | 0.313 | [0.287; 0.339] | 0.235 | [0.214; 0.255] |
| N+ | 0.190 | [0.169; 0.210] | 0.129 | [0.114; 0.143] |
| NX | 0.442 | [0.411; 0.473] | 0.286 | [0.264; 0.308] |
| **M-Stage** |  |  |  |  |
| M0 | 0.287 | [0.262; 0.312] | 0.208 | [0.189; 0.227] |
| M1 | 0.021 | [0.015; 0.028] | 0.014 | [0.009; 0.018] |
| MX | 0.637 | [0.600; 0.674] | 0.428 | [0.401; 0.455] |
| **Histological Subgroup** |  |  |  |  |
| Adenocarcinoma NOS | 0.174 | [0.155; 0.194] | 0.113 | [0.100; 0.127] |
| Mucoepidermoid carcinoma | 0.132 | [0.115; 0.149] | 0.105 | [0.091; 0.119] |
| Adenoid cystic carcinoma | 0.108 | [0.093; 0.123] | 0.081 | [0.069; 0.093] |
| Acinic cell carcinoma | 0.088 | [0.074; 0.102] | 0.073 | [0.061; 0.085] |
| Salivary duct carcinoma | 0.033 | [0.025; 0.042] | 0.023 | [0.017; 0.029] |
|  |  |  |  |  |
|  |  |  |  |  |
